# Cerebral hypoperfusion-related hemichorea: A case report and literature review

**DOI:** 10.1097/MD.0000000000042656

**Published:** 2025-06-06

**Authors:** Jia Shen, Quanzeng Zhang, Fanya Sun, Rong Li, Le Ding, Bo Zhu, Fang Yong, Zhiqin Liu, Naibing Gu, Zhengli Di

**Affiliations:** a Department of Neurology, Xi’an Central Hospital, Xi’an, Shaanxi, China.

**Keywords:** cardiac arrest, hemichorea, hypoxia, ischemia, stroke, transient ischemic attack

## Abstract

**Rationale:**

Hemichorea is an extrapyramidal disorder with diverse etiologies. Vascular hemichorea is most commonly caused by cerebral infarction in the putamen or caudate nucleus, with head diffusion weighted imaging (DWI) showing restricted diffusion at the site of the responsible lesion. Negative DWI in the basal ganglia is extremely rare. This article reports a case of hemichorea caused by a transient ischemic attack in the basal ganglia due to asymptomatic brief cardiac arrest, with negative basal ganglia DWI and dynamic electrocardiogram showing brief cardiac arrest (5.6 seconds).

**Patient concerns:**

An 82-year-old previously healthy female presented with “sudden slurred speech and involuntary right-sided body movements lasting 5 hours.”

**Diagnoses:**

DWI showed restricted diffusion in the left parieto-occipital lobe, but was negative in the basal ganglia. Dynamic electrocardiogram monitoring revealed atrial flutter and brief cardiac arrest (5.6 seconds). After antiplatelet aggregation therapy, the patient’s hemichorea completely resolved within 24 hours. Given the negative basal ganglia DWI and after excluding other causes, the diagnosis was a transient ischemic attack in the basal ganglia caused by the 5.6-second cardiac arrest.

**Interventions:**

Oral aspirin enteric-coated tablets and atorvastatin calcium tablets were administered, along with intravenous sodium dinitrophenyl chloride injection. The patient was then transferred to the cardiology department for implantation of a dual-chamber permanent pacemaker. Considering the patient’s atrial flutter and high bleeding risk, a long-term treatment plan with aspirin enteric-coated tablets and atorvastatin was selected.

**Outcomes:**

Symptoms completely resolved. During the 2-year follow-up, there was no recurrence or cerebrovascular disease-related events.

**Lessons:**

When patients present with acute hemichorea as the primary symptom and have negative DWI in the basal ganglia, a diagnosis of transient ischemic attack in the basal ganglia caused by asymptomatic brief cardiac arrest should be considered. The basal ganglia are particularly sensitive to ischemia, hypoxia, and reperfusion injury, which may be the mechanism by which brief cardiac arrest leads to hemichorea. Timely dynamic electrocardiogram monitoring helps clarify the diagnosis. Early implantation of a permanent pacemaker helps improve prognosis and prevent recurrence.

## 1. Introduction

Hemichorea is a common extrapyramidal motor disorder characterized by irregular, involuntary movements primarily affecting one side of the body, with acute cerebral infarction being the most frequent etiology and the lesions typically located in the basal ganglia. In recent years, it has been reported that cortical infarction can also cause hemichorea, but not involving the basal ganglia is rare.^[1,2]^ Cardiac arrest can cause global cerebral ischemia and hypoxia, and different areas within the brain have varying sensitivities to ischemia, hypoxia, and reperfusion injury following the restoration of blood flow, with the basal ganglia being the most sensitive to these ischemic and reperfusion injuries.^[2]^ Consequently, a brief cardiac arrest may lead to a transient ischemic attack in the basal ganglia, which could subsequently cause hemichorea. However, such cases are rarely reported currently. This article reports a case of unilateral chorea that was completely resolved within 24 hours. Diffusion weighted imaging (DWI) showed restricted diffusion in the left parieto-occipital lobe. Dynamic electrocardiography confirmed the presence of a 5.6-seconds sinus arrest and 47 beats per minute sinus bradycardia. After excluding various causes, we speculate that unilateral chorea is related to a transient ischemic attack (TIA) in the basal ganglia caused by the brief 5.6-second cardiac arrest. Combined with the literature analysis, the mechanism of unilateral chorea caused by cardiac arrest is emphasized, and the importance of timely antiplatelet aggregation, improving circulation, and correcting arrhythmias for prognosis is highlighted.

## 2. Case presentation

**Figure 1.**
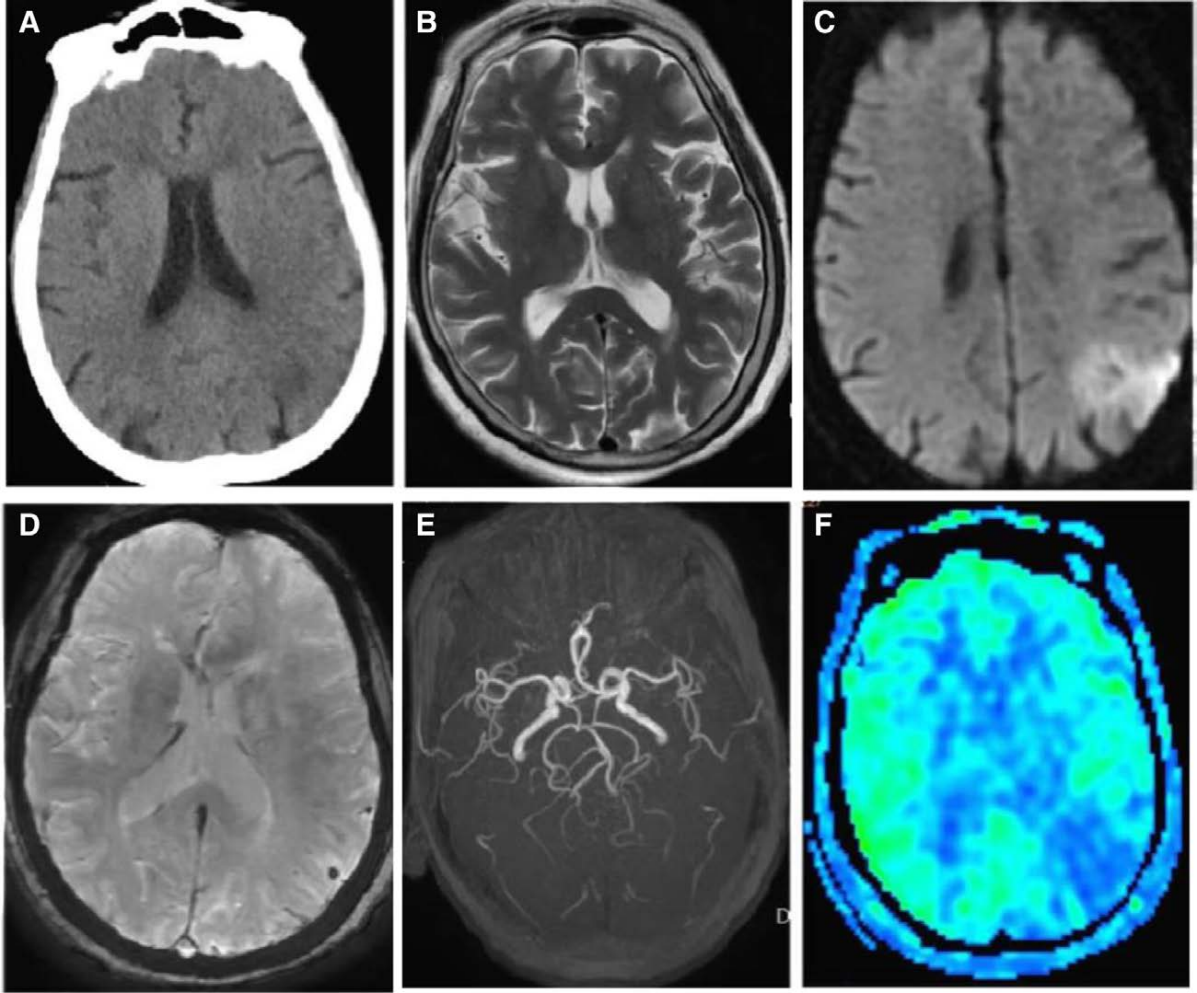
Patient’s imaging examination results. (A) Head CT showing lacunar cerebral infarction in the bilateral basal ganglia region. (B) Brain MRI showing cerebral infarction in the left parieto-occipital region. (C) Brain DWI showing high signal due to diffusion restriction in the left parieto-occipital region. (D) Brain SWAN showing microhemorrhage in the left parieto-occipital region. (E) Brain ASL showing reduced perfusion in the left temporal, parietal, and occipital lobes compared to the contralateral side. (F) Brain MRA showing a slightly narrow lumen of the right anterior cerebral artery. ASL = arterial spin labeling, CT = computed tomography, DWI = diffusion weighted imaging, MRI = magnetic resonance imaging.

A previously healthy 82-year-old female presented with “sudden slurred speech and involuntary right-sided body movements lasting 5 hours” (see Video S1, Supplemental Digital Content, https://links.lww.com/MD/P52, which demonstrates right-sided body movements at the time of admission). Neurological examination revealed sluggish speech and slow responses. The right nasolabial fold was shallow and the tongue was deviated to the right. There was spontaneous, continuous, and irregular involuntary movement of the right limb with decreased muscle tone, with no other significant findings in the neurological examination. Fasting blood glucose was 4.63 mmol/L (3.9–6.1 mmol/L), and glucose tolerance test showed impaired glucose tolerance. Other routine serum chemistry tests showed normal results. A 24-hour Holter monitoring showed paroxysmal atrial flutter, ventricular premature beats, and brief cardiac arrest (5.6 seconds). Echocardiogram showed an EF of 46% and a FS of 21%. The video electroencephalogram monitoring was normal. Head computed tomography (CT) showed lacunar cerebral infarction in the bilateral basal ganglia region (Fig. 1A). Brain magnetic resonance imaging (MRI) showed fresh cerebral infarction with microhemorrhage in the left parieto-occipital lobe; reduced blood flow perfusion in the left temporal, parietal, and occipital lobes compared to the contralateral side; and slightly narrow lumen of the right anterior cerebral artery (Fig. 1B–F). After antiplatelet therapy, the unilateral chorea completely resolved within 24 hours (see Video S2, Supplemental Digital Content, https://links.lww.com/MD/P53, which demonstrates the complete absence of involuntary movement of the right-sided body).

The patient is an elderly female who presented with an acute onset of symptoms, primarily characterized by unclear speech and chorea-like movements of the right limbs. DWI revealed restricted diffusion in the left parieto-occipital region, but no responsible lesions were found in the basal ganglia. Holter monitoring showed a brief cardiac arrest lasting 5.6 seconds. Serological tests showed no related abnormalities. After ruling out various causes, the final diagnoses were: (1) TIA, (2) cerebral infarction, and (3) cardiac arrest. The patient experienced no discomfort during dynamic electrocardiogram monitoring. After the electrocardiogram department reported a 5.6-second cardiac arrest in the patient, an immediate cardiology consultation was requested. Since there were no ventricular arrhythmias or hemodynamic abnormalities, the cardiologist decided to implant a dual-chamber permanent pacemaker. Although the patient had atrial flutter, long-term treatment with enteric-coated aspirin tablets and atorvastatin calcium tablets was chosen due to the high risk of bleeding. During the 2-year follow-up after discharge, there was no recurrence, no cerebrovascular events, and no evidence of carotid artery occlusion or stenosis, which further supports the current diagnosis.

## 3. Discussion

Hemichorea, characterized by spontaneous, irregular, and purposeless limb or facial movements, is a common extrapyramidal motor disorder. The known causative factors include vascular, metabolic, neoplastic, infectious, autoimmune, and genetic etiologies. Acute stroke typically presents with sudden limb paralysis, facial palsy, aphasia, and disturbances of consciousness, while poststroke movement disorders are less common.^[1]^ It has been reported that hemichorea following stroke typically occurs within 24 hours after the stroke, with lesions often located in the putamen and caudate nucleus. The caudate nucleus and putamen together form the neostriatum, which is part of the cortico-striatal-thalamo-cortical neural circuit that is crucial for motor coordination. Lesions in any part of this circuit may lead to hemichorea.^[3]^ In recent years, there have been multiple reports of hemichorea caused by cortical infarctions in the frontal, parietal, or occipital lobes, but in most cases, the DWI restricted diffusion areas involve the basal ganglia, and there are few reports of negative DWI cases in the basal ganglia region.^[4,5]^ A case of a parietal infarction patient with no basal ganglia lesions on head DWI presented with hemichorea, and single-photon emission CT showed hypoperfusion in the basal ganglia, indicating that ischemia in the basal ganglia region can lead to functional imbalance and result in hemichorea.^[6]^ Contrary to this, Murakami et al also reported a case of a patient with temporo-parietal infarction who had no basal ganglia lesions on head DWI and developed hemichorea after rtPA thrombolysis with improved hemiplegia. However, SPECT showed an increase in blood flow in the right basal ganglia, which may be due to reperfusion injury caused by increased blood flow in the basal ganglia leading to hemichorea.^[7]^ These indicate that both ischemia and reperfusion injury in the basal ganglia can lead to functional imbalances, resulting in hemichorea.

The pathological process of global cerebral ischemia and hypoxia caused by cardiac arrest, and reperfusion injury after blood flow recovery, is roughly divided into 2 stages: first, injury caused by ischemia–hypoxia; second, injury caused by ischemia–hypoxia, with the extent of injury varying depending on the area of the brain.^[8]^ The brain’s neuronal activity relies on a continuous supply of oxygen and energy substrate.^[9]^ Neurons in layers III, V, and VI of the cerebral cortex are the destinations for the majority of cortico-cortical and thalamo-cortical afferents and are crucial for arousal and consciousness, being the most sensitive to ischemic and hypoxic damage. Similarly susceptible neurons are found in the hippocampal CA1 region, the caudate nucleus, the putamen, the globus pallidus, certain brainstem nuclei, and Purkinje cells in the cerebellar cortex, which may be the reason why these areas are prone to neuronal damage.^[10]^ Furthermore, the reperfusion following transient global cerebral ischemia induced by cardiac arrest is incomplete and uneven. Histologically, this is manifested as multiple perfusion defects, which are more commonly seen in the striatum, thalamus, hippocampus, and amygdala. Animal studies have indicated that the basal ganglia, hippocampus, and thalamus are among the regions most severely affected by reperfusion injury following cardiac arrest, with the extent of damage significantly correlated to the time it takes for the heart to regain spontaneous circulation.^[11,12]^ There have been reports of patients who remained comatose due to cardiac arrest exhibiting involuntary limb movements. Two months later, the patient’s brain MRI showed lesions in the bilateral caudate nucleus, putamen, thalamic nuclei, and substantia nigra.^[13]^ All in all, due to the high sensitivity of the basal ganglia region to ischemia and hypoxia, it is the most common site for reperfusion injury following cardiac arrest. Therefore, even a brief cardiac arrest may lead to transient ischemic attacks and reperfusion injury in the basal ganglia region, thereby triggering hemichorea.

The localization diagnosis of the patient’s choreic movements on the right side does not match the newly developed watershed cerebral infarction in the left parieto-occipital lobe as shown by DWI; Symptoms completely resolved within 24 hours of onset; video electroencephalogram and serum biochemical tests showed no relevant abnormalities. Thus, even though the cardiac arrest in this patient was brief at 5.6 seconds as monitored by Holter monitoring, the lateral chorea is likely associated with a TIA in the basal ganglia due to the ischemia and hypoxia sensitivity of this region following the cardiac arrest. However, this patient also had atrial flutter, which could potentially lead to thrombotic events, but according to the neurovascular anatomy, it is less likely for a thrombus to cause both a watershed cerebral infarction in the parieto-occipital lobe and a transient ischemic attack in the basal ganglia simultaneously. In conclusion, transient cardiac arrest leading to transient ischemic attack in the basal ganglia, which is sensitive to ischemia and hypoxia, is possible. During the 2-year follow-up after discharge, the patient had no recurrence of hemichorea and no cerebrovascular events, which further supported our diagnosis. Although this case explores the potential mechanism by which cardiac arrest leads to TIA in the basal ganglia, thereby triggering hemichorea, the current sample size is limited, and more cases are needed for follow-up observation and validation. Furthermore, future studies that incorporate multimodal magnetic resonance techniques, such as SPECT or PET, to assess the impact of cardiac arrest on the brain will help to further investigate the pathophysiological mechanisms. In summary, for patients with acute hemichorea, timely and comprehensive dynamic electrocardiogram monitoring can provide important clues for identifying potential causes.

## 4. Conclusion

In cases where patients exhibit sudden-onset lateral chorea as the primary symptom, aside from the usual suspects of metabolic, neoplastic, autoimmune, genetic, and infectious diseases, one should also contemplate the likelihood of silent brief cardiac arrest or atrial fibrillation causing transient ischemic attacks in the basal ganglia. Holter monitoring is conducive to early diagnosis and treatment, which is advantageous for enhancing patient prognosis and preventing relapses.

## Acknowledgments

We would like to thank all the clinicians providing care and management to the patients.

## Author contributions

**Formal analysis:** Le Ding.

**Writing – original draft:** Jia Shen, Quanzeng Zhang, Fanya Sun, Rong Li, Bo Zhu.

**Writing – review & editing:** Quanzeng Zhang, Fang Yong, Naibing Gu, Zhiqin Liu, Zhengli Di.

## Supplementary Material



## References

[1] Defebvre L Krystkowiak P. Movement disorders and stroke. Rev Neurol (Paris). 2016;172:483–7. 27476417 10.1016/j.neurol.2016.07.006

[2] Kwon DY. Movement disorders following cerebrovascular lesions: etiology, treatment options and prognosis. J Mov Disord. 2016;9:63–70. 27240807 10.14802/jmd.16008 PMC4886206

[3] Miletić V Blažina K. Hemidystonia caused by frontal cortical infarction. Acta Neurol Belg. 2015;115:383–4. 25119274 10.1007/s13760-014-0354-3

[4] Carbayo A Sarto J Santana D Compta Y Urra X. Hemichorea as presentation of acute cortical ischemic stroke. Case series and review of the literature. J Stroke Cerebrovasc Dis. 2020;29:105150. 32912504 10.1016/j.jstrokecerebrovasdis.2020.105150 PMC7384777

[5] Salgado P Taipa R Domingos J Dias D Pires MM Magalhães M. Vascular pathology causing late onset generalized chorea: a clinico-pathological case report. Mov Disord Clin Pract. 2017;4:819–23. 30363429 10.1002/mdc3.12528 PMC6174401

[6] Mizushima N Park-Matsumoto YC Amakawa T Hayashi H. A case of hemichorea–hemiballism associated with parietal lobe infarction. Eur Neurol. 1997;37:65–6. 9018037 10.1159/000117408

[7] Murakami T Wada T Sasaki I. Hemichorea–hemiballism in a patient with temporal–parietal lobe infarction appearing after reperfusion by recombinant tissue plasminogen activator. Mov Disord Clin Pract. 2015;2:426–8. 30838242 10.1002/mdc3.12198 PMC6353402

[8] Sandroni C Cronberg T Sekhon M. Brain injury after cardiac arrest: pathophysiology, treatment, and prognosis. Intensive Care Med. 2021;47:1393–414. 34705079 10.1007/s00134-021-06548-2 PMC8548866

[9] Annoni F Peluso L Gouvêa Bogossian E Creteur J Zanier ER Taccone FS. Brain protection after anoxic brain injury: is lactate supplementation helpful? Cells. 2021;10:1714. 34359883 10.3390/cells10071714 PMC8305209

[10] Babikian VL Caplan LR. Brain embolism is a dynamic process with variable characteristics. Neurology. 2000;54:797–801. 10690965 10.1212/wnl.54.4.797

[11] Suri R Rodriguez-Porcel F Donohue K. Post-stroke movement disorders: the clinical, neuroanatomic, and demographic portrait of 284 published cases. J Stroke Cerebrovasc Dis. 2018;27:2388–97. 29793802 10.1016/j.jstrokecerebrovasdis.2018.04.028

[12] Haglund M Lindberg E Englund E. Hippocampus and basal ganglia as potential sentinel sites for ischemic pathology after resuscitated cardiac arrest. Resuscitation. 2019;139:230–3. 31005590 10.1016/j.resuscitation.2019.04.012

[13] Di Lazzaro V Pilato F Saturno E. Bilateral chorea–ballism after cardiac arrest. Neurology. 2005;64:E20. 15781800 10.1212/wnl.64.6.e20

